# Evaluation of the antibacterial activity of Enamelast® and Fluor defender® fluoride varnishes against *Streptococcus mutans* biofilm: an in vitro study in primary teeth

**DOI:** 10.1007/s40368-023-00811-4

**Published:** 2023-08-01

**Authors:** M. A. Matar, S. S. Darwish, R. S. Salma, W. A. Lotfy

**Affiliations:** 1 Pediatric and Community Dentistry Department, Faculty of Dentistry, Pharos University in Alexandria, Alexandria, Egypt; 2 Pediatric Dentistry Department, College of Dentistry El Alamein, Arab Academy for Science, Technology and Maritime Transport (AAST), Alamein, Egypt; 3 Microbiology Department, Faculty of Dentistry, Pharos University in Alexandria, Alexandria, Egypt

**Keywords:** Antimicrobial, Fluoride varnish, *Streptococcus mutans*, Caries

## Abstract

**Purpose:**

The aim of the current work was to compare the antibacterial activity of Enamelast® and Fluor defender® fluoride varnish on biofilm generation by *Streptococcus mutans* on extracted primary teeth.

**Methods:**

Thirty-six primary molars were collected and sliced into seventy-two test model disks. All specimens were examined, and the cracked or broken ones were discarded. A total number of specimens (*n* = 54) were divided into two experimental analyses viz; biofilm formation (*n* = 27) and microscopic examination (*n* = 27). Specimens of each analysis were tested under different experimental conditions: a negative control group (*n* = 9), Fluor defender group (*n* = 9), and Enamelast group (*n* = 9). Following treatment, biofilms were generated by adherent *Streptococcus mutans* on the test model disks on three time intervals: 24 h (*n* = 3), 48 h (*n* = 3), and 72 h (*n* = 3) for each analysis. Then, for biofilm formation analysis, the biofilm was detected spectrophotometrically at 620 nm after being stained by crystal violet. For microscopical analysis, the surfaces of the test model disks were visualized by scanning electron microscopy (SEM), and each image was processed and analyzed using ImageJ software.

**Results:**

At 48 and 72 h, Enamelast® and Fluor defender®-treated group showed significantly (*p* < 0.001) slight adhered bacterial cells when compared with the negative control group as revealed by the absorbance and SEM. Compared with the Fluor defender®-treated group, the absorbance of the Enamelast®-treated group showed a significant (*p* < 0.001) increase by approximately 7- and 16.5-fold at 48 and 72 h, respectively. Similarly, SEM showed that the number of bacterial cells adhered to enamel surfaces in the Fluor defender®-treated group was significantly (*p* < 0.001) fewer than the Enamelast®-treated group by approximately 36.55% and 20.62% at 48 and 72 h after exposure, respectively.

**Conclusion:**

We conclude that the anti-biofilm activity of Fluor defender® against *Streptococcus mutans* was significantly (*p* < 0.001) greater than Enamelast® fluoride varnish. The use of Fluor defender® is encouraged as a preventive measure in children with the high risk of developing dental caries.

## Introduction

Dental caries is a chronic, multifactorial, bacterial disease causing enamel demineralization and disintegration of the organic substances of the teeth (Karpiński and Szkaradkiewicz 2013). The etiology of caries includes host factors, carbohydrates intake, plaque bacteria, and time (Samaranayake 2018). A homeostasis occurs between demineralization and remineralization. However, if this balance is disturbed, demineralization overtakes remineralization leading to dental caries (García-Godoy and Hicks 2008; Salma et al. 2022; Stephan and Miller 1943; Takahashi and Nyvad 2008). Biofilm plays an essential role in the initiation and progression of dental caries (Lee et al. 2010). It metabolizes dietary carbohydrates via glycolysis to form lactic acid leading to a drop in pH levels and, consequently, causing enamel demineralization (Pandit et al. 2015).

Acidogenicity and acidurity are considered crucial factors for the survival of biofilm (Pandit et al. 2013). Another key feature is the ability of biofilm to synthesize water-insoluble glucans from glucose by the enzyme glucosyltransferases (Pandit et al. 2013). *Streptococcus mutans* has both acidogenic and aciduric characteristics. Therefore, it is identified as the primary source of caries initiation (ten Cate 2006). Lactic acid is produced by *Streptococcus mutans* through fermentation of dietary carbohydrates. Drop in oral pH contributes to both dominance of the *Streptococcus mutans* and formation of caries. Demineralization occurs by a complex interaction between commensals, carbohydrates, and salivary components. Demineralization overtakes remineralization when the pH at the enamel surface drops below 5.5 (Loesche 1986).

Recently, early childhood caries (ECC) has become a considerable public health problem (Anil and Anand 2017; Çolak et al. 2013). Despite the decrease in dmft index in the developed countries, it is increasing in the developing world nations (Anil and Anand 2017; Folayan et al. 2020). One of the most essential factors that predispose ECC is the formation of acidogenic and aciduric biofilm of Mutans Streptococci (Carlsson 1997; Ccahuana-Vásquez and Cury 2010; Hamada et al. 1984; Hamada and Slade 1980; Seow 1998). Therefore, biofilm control is crucial for prevention of ECC. Fluoride application is one of the main strategies used to control ECC by enhancing remineralization (Cate and Featherstone 1991), preventing demineralization (Tenuta et al. 2009), and induction of anti-biofilm activities of tooth enamel (Pandit et al. 2013). Fluoride accelerates the remineralization process by adsorbing to the enamel surface and attracting phosphate and calcium ions. Additionally, fluoride substitutes the hydroxyl ions in hydroxyapatite of enamel forming fluorapatite which has greater resistance to bacterial acids (Featherstone 1999).

Many companies are globally striving to develop fluoride varnish that can adhere to the tooth surface to improve the antibacterial properties and acid resistance. Cerkamed Co., Poland, has developed Fluor defender® that comprises hydroxyethyl methacrylate which contains 0.1% fluorosilane. Fluor defender® can be used to improve remineralization, strength of enamel, and to build a protective layer on the enamel’s surface. Enamelast®, a product of Ultradent Co., USA, is a flavored, xylitol-sweetened 5% sodium fluoride in a resin carrier which produces a mechanical occlusion of the dentinal tubules in the treatment of tooth hypersensitivity. However, to the best of our knowledge, there are no reports that investigate the antibacterial activities of Fluor defender® or Enamelast® on primary teeth enamel. The aim of the current study is to evaluate the effect of Enamelast® on the formation of *Streptococcus mutans* biofilm, as compared to Fluor defender® on primary teeth. The null hypothesis tested in this study was that Enamelast® varnish has the same antibacterial efficacy of Fluor defender® varnish on the formation of *Streptococcus mutans* biofilm.

## Materials and methods

### Study design, setting, and ethical consideration

This was an in vitro study. The study protocol was reviewed and approved by the Research Ethical Committee, Pharos University in Alexandria (# PUA02202208283041). It  was in accordance with The Code of Ethics of Pharos University in Alexandria for experiments involving human subjects. The procedures used in this study adhere to the tenets of the Declaration of Helsinki. A written informed consent was acquired from the parents of the subjects before donation their shedding sound primary teeth.

**Fig. 1:**
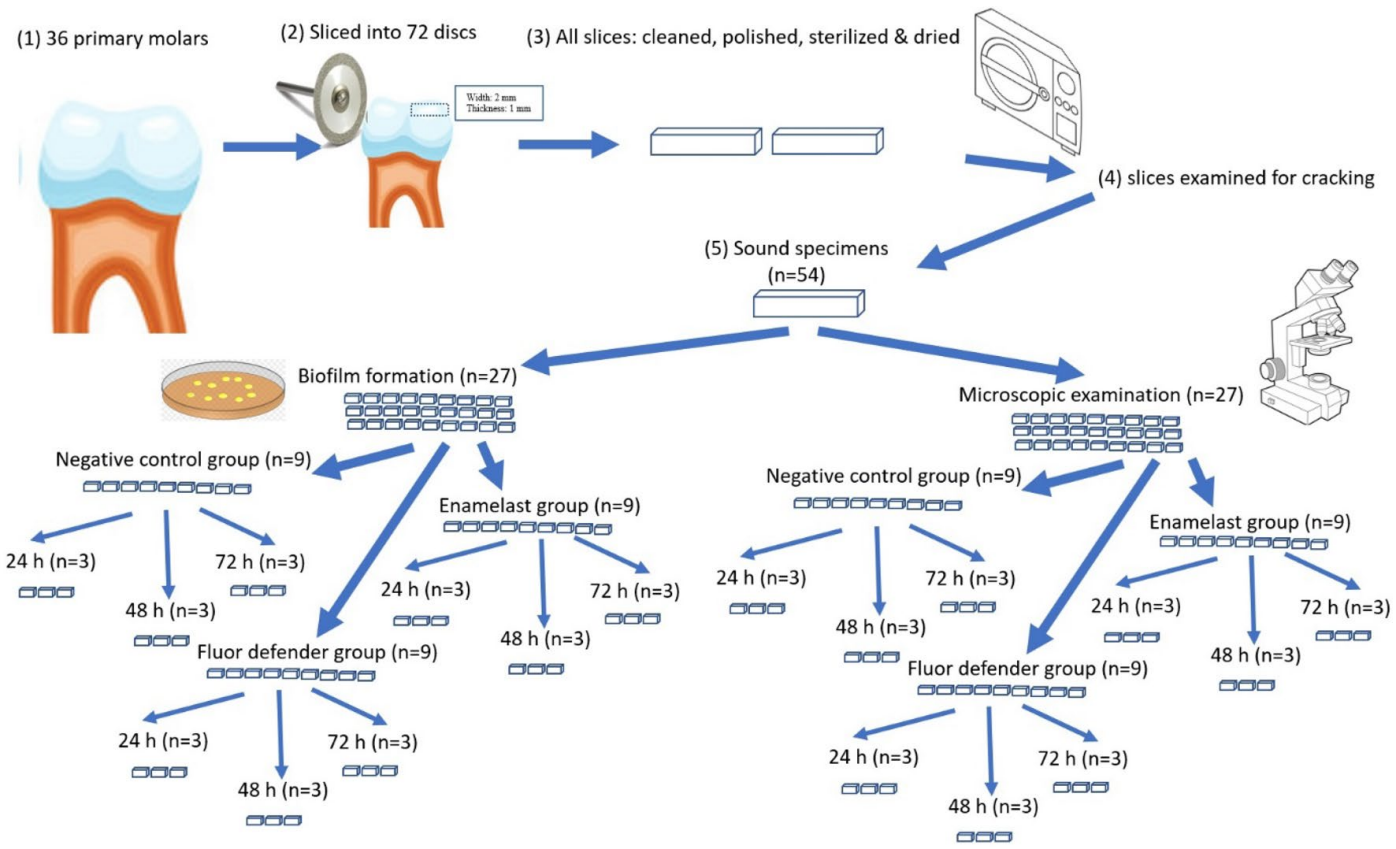
Diagram of the sample processing and distribution

The minimal sample size was calculated to a total of 24 specimens divided into 3 groups with a sample size of 8 per group and 2.38 per subgroup according to the following equation:

$$n= \frac{2({Z}_{a}+{Z}_{1-\beta }){2\sigma }^{2}}{{\Delta }^{2}}$$

where n is the required sample size. For *Z*_*α*_, *Z* is a constant set by convention according to the accepted *α* error. For *Z*_1-β_ , *Z* is a constant set by convention according to power of the study. *σ* is the standard deviation and Δ is the difference in effect of two interventions. The number of specimens per group was increased to 9 to make the specimens equal in number through all 3 intervals. Thirty-six primary molars were collected from the out-patient clinic of the Pediatric Dentistry department, and then sliced into seventy-two test model disks with a width of 2 mm and a thickness of 1 mm. The surface of each disk was cleaned, polished, sterilized in an autoclave, and dried with air stream. All specimens were examined, and the cracked or broken ones were discarded. A total number of specimens (*n* = 54) were randomly divided into two experimental analyses, namely biofilm formation (*n* = 27) and microscopic examination (*n* = 27), by a computerized random sequence generator. Next, specimens of each analysis were randomly assigned into a negative control group (*n* = 9) where no processing was applied, Fluor defender group (*n* = 9) where it was applied as specified by the manufacturer, and Enamelast group (*n* = 9) where Enamelast was applied as specified by the manufacturer. In both analyses and for all groups, the specimens were allocated randomly to one of the three time intervals: 24 h (*n* = 3), 48 h (*n* = 3), and 72 h (*n* = 3). A thin layer of Fluor defender or Enamelast was applied on the enamel surface using brush applicator then dried with air stream for 30 s (Fig. 1).

### Chemicals and dehydrated media

Tryptone soya broth (HiMedia Laboratories, India) was used for biofilm formation. Media were prepared according to the manufacturer’s instructions before autoclaving at 121 °C for 15 min. All chemicals used throughout the current study were of analytical grade. Sucrose was a product of Loba Chemie, India. Fluor defender (0.1% fluorosilane equivalent to 1600 ppm fluoride) is a fluoride varnish produced by Cerkamed Co., Poland. Enamelast® (5% sodium fluoride equivalent to 22,600 ppm fluoride) is a product of Ultradent Co., USA.

### Microorganisms

The bacterium used throughout this work was *Streptococcus mutans* (ATCC 25175).

### Biofilm formation on test model disks

*Streptococcus mutans* was inoculated in tryptone soya broth media, and incubated under anaerobic conditions at 37 °C. After cultivation, bacterial suspensions of *Streptococcus mutans* were adjusted to 0.5 McFarland using sterile saline. Biofilm of *Streptococcus mutans* was generated in sterile 24-well plate, each well received a tooth test model, 1 ml tryptone soya broth media containing 0.5% sucrose, and 10 μl bacterial suspension. The plates were incubated anaerobically at 37 °C until a biofilm was formed on the disk surface (Jafri et al. 2019).

### Detection of biofilm by crystal violet staining

For reproducibility, three test model disks were collected from each group at 24 h, 48 h, and 72 h. The disks were washed three times with sterile phosphate-buffered saline (PBS) to exfoliate non-adherent bacterial cells. The disks were allowed to dry prior staining with 200 μl crystal violet (0.4%) for 15 min. The disks were washed three times with PBS then air-dried for 15 min. A volume of 200 μl acetic acid (33%) was used to dissolve the residual crystal violet in each disk. A microplate reader (MR-96, Clindiag Systems Co. LTD., China) was used to measure the absorbance of the solution at 620 nm (Stepanović et al. 2000).

### Detection of biofilm by scanning electron microscopy (SEM)

Three test model disks were collected from each group at 24 h, 48 h, and 72 h. Each disk was washed three times with sterile PBS to shed non-adherent bacterial cells. Test model disks with adherent biofilms were fixed with 2.5% glutaraldehyde in a series of PBS solution. Subsequently, the disks were washed with sterile distilled water and dehydrated with ethanol (Lotfy et al. 2021). The surfaces of the test model disks were visualized by SEM (JSM-IT700HR, JEOL Co., Ltd., Japan). After capturing images by SEM, each image was processed and analyzed using ImageJ software, and the number of bacterial cells was calculated as average of 3 fields from each test model disk (Schneider et al. 2012). To ensure reproducibility, the analysis was validated by standardized bacterial concentration inoculated on the surface of three test model disks.

### Statistical analysis

Statistical analysis of the results was performed by applying one-way ANOVA test using a significance threshold of *p* < 0.001 for the 3 groups (*n* = 27) per each analysis.

## Results

### Detection of biofilm by crystal violet staining following experimental treatment

**Fig. 2:**
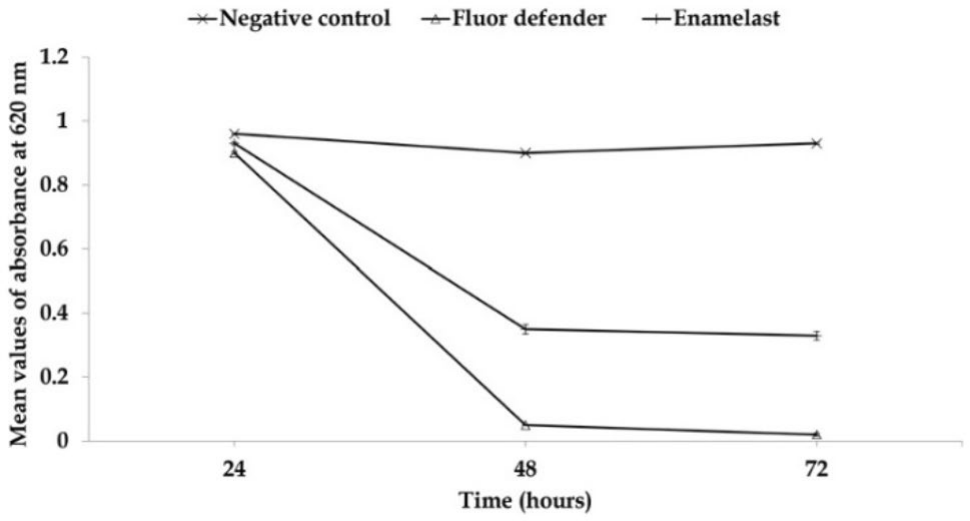
The effect of Fluor defender® and Enamelast® on *Streptococcus mutans* biofilm compared to negative control

No significant difference was observed in the absorbance of residual crystal violet stain at 620 nm in any of three groups up to 24 h following experimental treatment (Table 1 and Fig. 2). On the other hand, a significant difference was observed between the negative control group and the Enamelast®-treated group at 48 and 72 h (*p* < 0.001) as shown in Table 1 and Fig. 2. Similarly, the Fluor defender®-treated group was significantly different from the negative control group (*p* < 0.001) (Table 1 and Fig. 2). However, after 48 and 72 h, the absorbance from the Enamelast®-treated experimental group was significantly (*p* < 0.001) greater than that of Fluor defender® by 7- and 16.5-fold increase, respectively (Table 1 and Fig. 2).

**Table 1:**
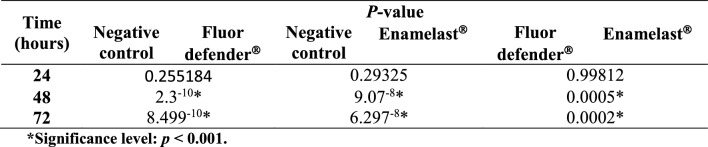
ANOVA *p*-value of negative control, Fluor defender®, and Enamelast® groups with respect to the absorbance of residual crystal violet following experimental treatment

*Significance level: *p* < 0.001

### Detection of biofilm by SEM

**Fig. 3:**
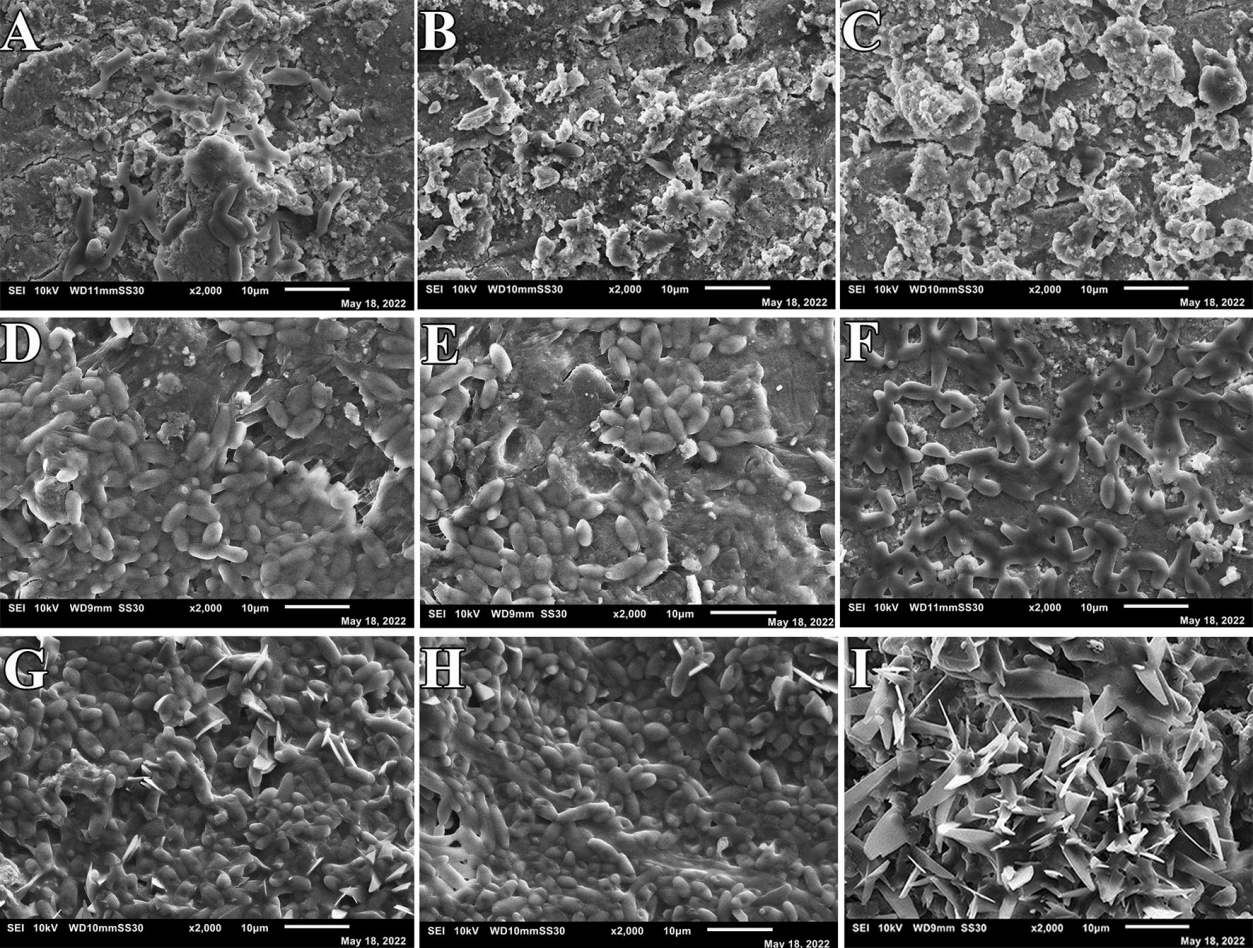
SEM (2000 ×) of negative control at 24 h (**A**–**C**), Fluor defender®at 24 h (**D**–**F**), and Enamelast® at 24 h (**G**–**I**)

**Fig. 4:**
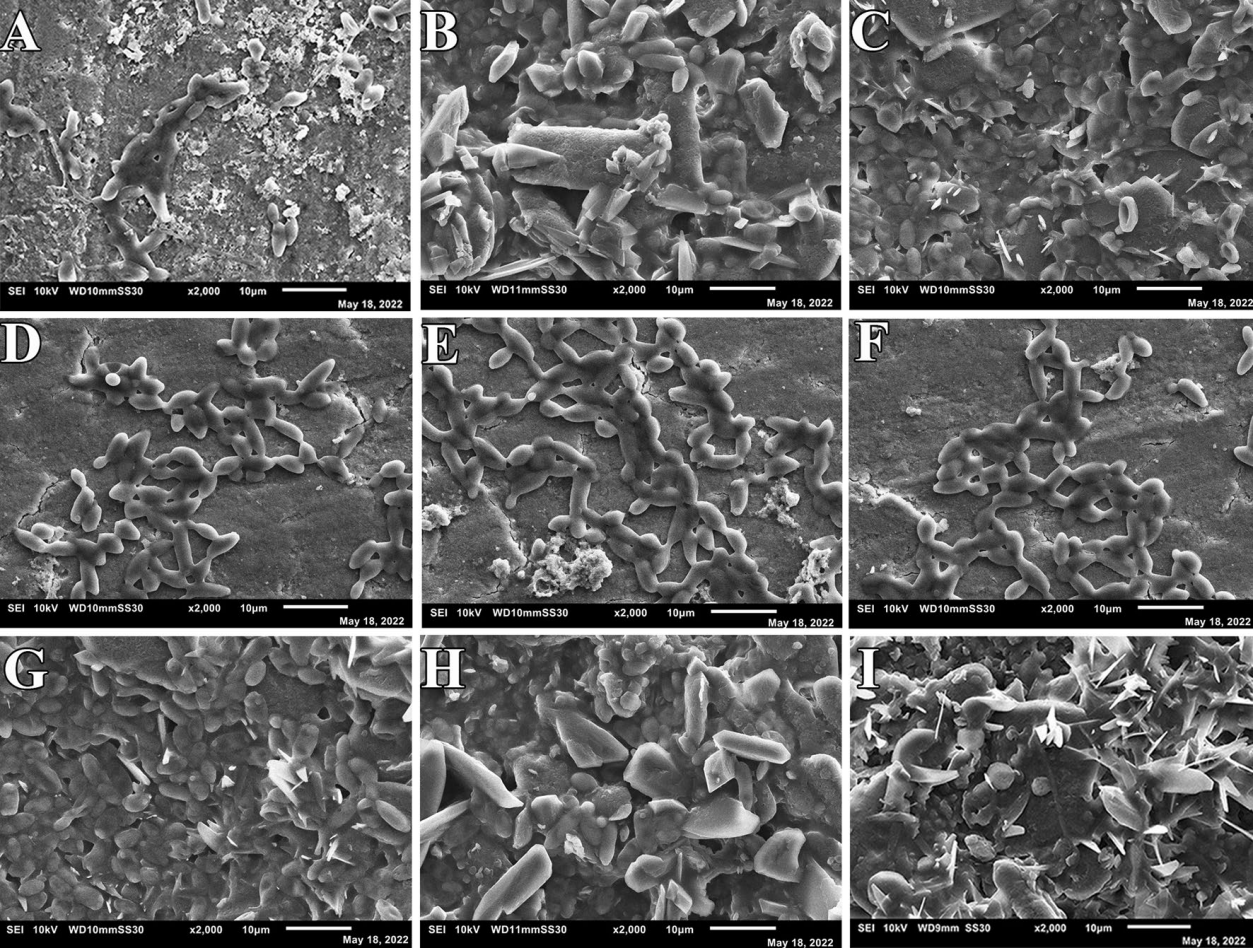
SEM (2000 ×) of negative control at 48 h (**A**–**C**), Fluor defender® at 48 h (**D**–**F**), and Enamelast® at 48 h (**G**–**I**)

**Fig. 5:**
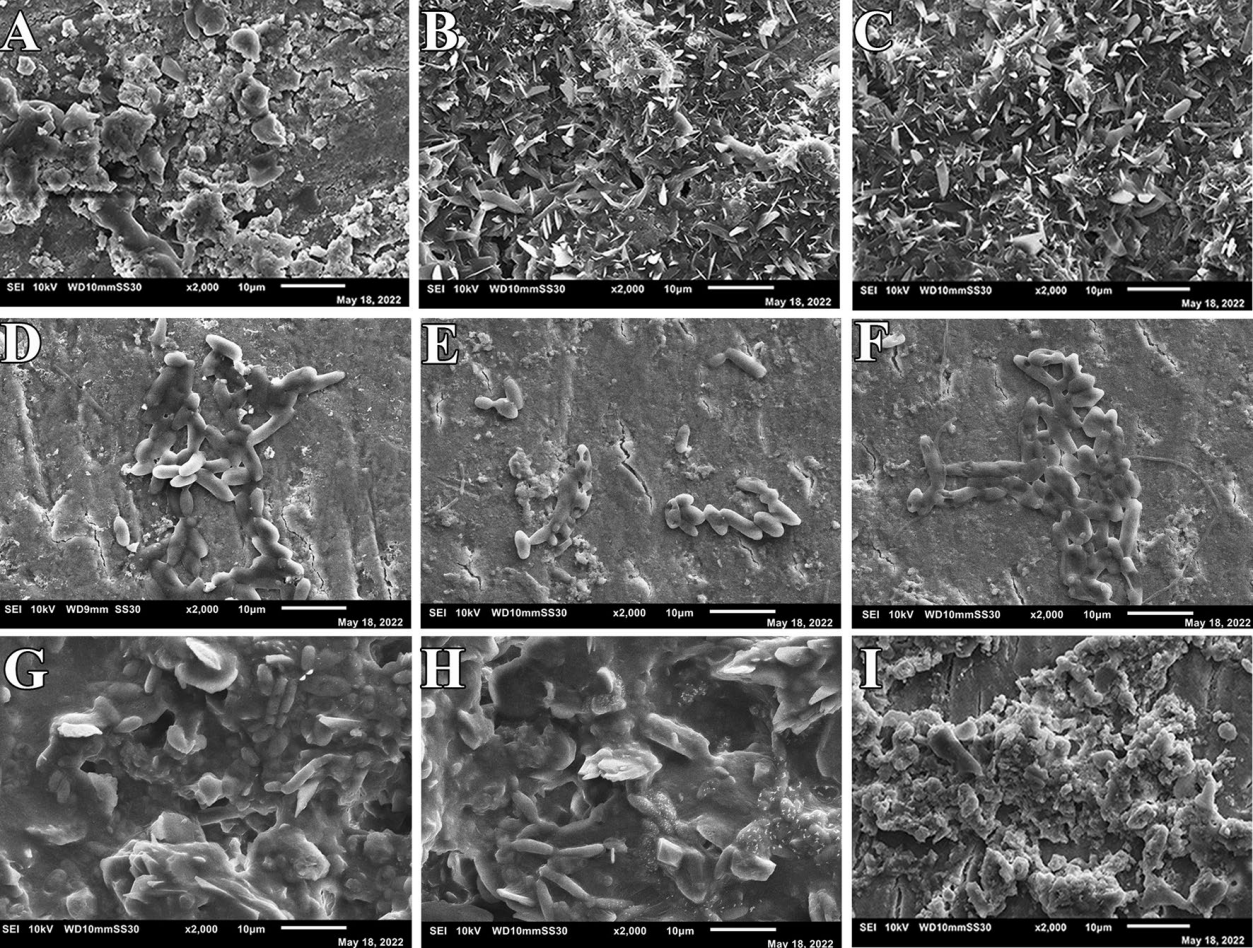
SEM (2000 ×) of negative control at 72 h (**A**–**C**), Fluor defender® at 72 h (**D**–**F**), and Enamelast® at 72 h (**G**–**I**)

**Fig. 6:**
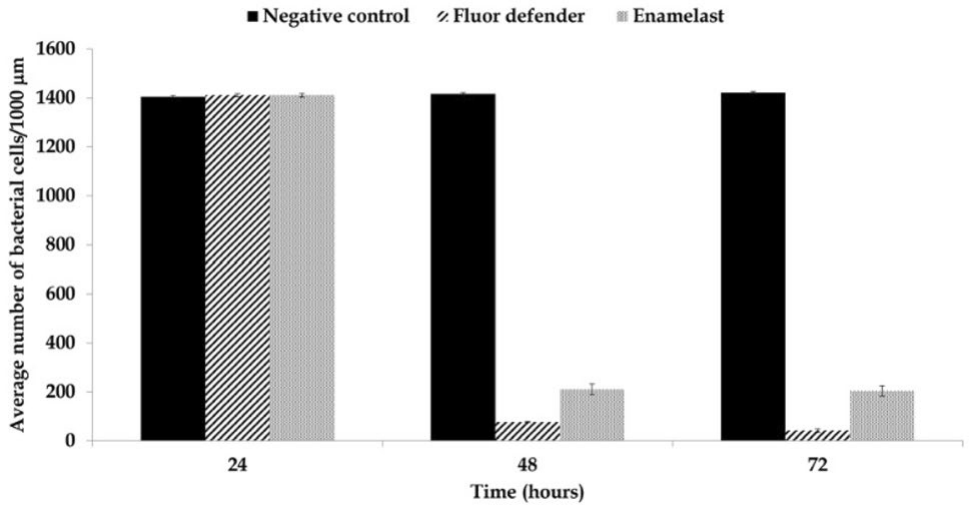
Average number of *Streptococcus mutans* cells on test disk models of negative control, Fluor defender®, and Enamelast® groups as revealed by SEM image analysis

At 24 h, the biofilm formed by *Streptococcus mutans* on the enamel surface was visible in the negative control group, Fluor defender®-treated group, and Enamelast®-treated group (Fig. 3). Furthermore, the biofilms of *Streptococcus mutans* under the SEM appeared as an extensible structure overlaying each test model surface of the three groups (Fig. 3). Moreover, no significant difference was observed in the number of *Streptococcus mutans* cells formed by adhering to test surfaces in any of three groups (*p* < 0.001) (Table 2). At 48 and 72 h, both the Fluor defender®-treated group and Enamelast®-treated group demonstrated only scattered *Streptococcus mutans* cells with no extensible structures (Figs. 4 and 5). However, the negative control group showed extensible structures of glomerated biofilm at 48 and 72 h (Figs. 4 and 5). After 48 h, the number of *Streptococcus mutans* cells adhered to enamel surfaces in the Fluor defender®-treated experimental group was significantly (*p* < 0.001) fewer than the Enamelast®-treated group by approximately 36.55% (Table 2 and Fig. 6). Similarly, after 72 h, the number of *Streptococcus mutans* cells remained on the enamel surfaces in the Enamelast®-treated experimental group was significantly (*p* < 0.001) greater than the Fluor defender®-treated group by approximately 79.38% (Table 2 and Fig. 6).

**Table 2:**
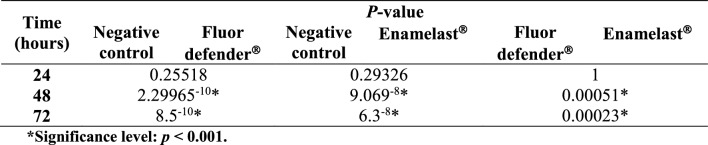
ANOVA *p*-value of negative control, Fluor defender®, and Enamelast® groups with respect to the number of *Streptococcus mutans* cells on test disk models

## Discussion

Virulence attributes of biofilm sheathed bacteria, such as acidogenicity, acidurity, and formation of extracellular polysaccharides, induce an acidic microenvironment that causes ecological dysbiosis (Pandit et al. 2013; Philip et al. 2018). Subsequently, a shift in the homeostasis of oral bacteria in favor of cariogenic flora occurs which predisposes the teeth to dental caries (Schwendicke et al. 2016). Removal of dental plaque biofilm by mechanical cleansing is an effective means to disrupt the caries process. However, reformation of bacterial biofilm starts immediately afterward.

Enamel remineralization has been suggested as a non-invasive treatment of ECC by remineralization in the clinical management of the disease (Shen et al. 2011). This takes place when the pH rises and phosphate, calcium, and fluoride ions deposit on tooth enamel in the form of fluorapatite which is more resistant to organic acids than hydroxyapatite (Cilurzo et al. 2003). Many investigations have tested the efficacy of antimicrobials against biofilms cariogenicity (Dang et al. 2016; Kulshrestha et al. 2016; Pandit et al. 2013). Fluoride has been reported as the gold standard agent for caries control (Zero 2006). To date, no studies have focused on comparing the antibacterial efficacy of Enamelast® and Fluor defender® on biofilm formation of *Streptococcus mutans*. Hence, our aim was to evaluate the effect of these fluoride varnishes on the formation of *Streptococcus mutans* biofilm.

In the current study, Fluor defender® and Enamelast® were applied to enamel tooth surfaces of primary teeth. Thenceforward, the biofilm formation was detected spectrophotometrically and was observed by SEM in order to investigate the anti-biofilm activity of Fluor defender® and Enamelast®. Primary teeth specimens were used in the current study for biofilm growth. Although bovine enamel has been used in many studies (Lippert and Lynch 2014), using human enamel specimens is more clinically relevant. The growth medium used was tryptone soya broth, supplemented with sucrose to maintain the viability of *Streptococcus mutans*. The same medium was documented in previous studies (Latimer et al. 2015; Lotfy et al. 2018; Zhang et al. 2015).

Compared with the negative control group at 48 and 72 h, Enamelast® and Fluor defender®-treated group showed significantly (*p* < 0.001) slight adhered bacterial cells as revealed by the absorbance and SEM as well. This emphasizes the antimicrobial effect of both types as attributed to fluoride content which interfered with bacterial metabolism and inhibited bacterial growth (Bradshaw et al. 2002). However, no significant difference was observed in the bacterial adherence in any of three groups up to 24 h following experimental treatment. In this context, we emphasize that tooth-brushing behavior should not be carried out at least 24 h following the application of fluoride varnish to avoid reducing the amount of attached varnish on teeth surfaces.

The fluoride varnishes, Fluor defender®, and Enamelast® were able to protect the under-treatment area against biofilm formation by *Streptococcus mutans*. Although Enamelast® has an enhanced retention on the tooth surface allowing higher fluoride uptake (Godoi et al. 2019). Nevertheless, the absorbance from the Enamelast®-treated group, respectively, showed 7- and 16.5-fold increase up to 48 and 72 h after exposure when compared to the Fluor defender®-treated group (*p* < 0.001). Moreover, in the SEM images, there were visibly fewer cells of *Streptococcus mutans* attached to the enamel surfaces from 48 to 72 h after exposure to Fluor defender® than Enamelast®. The number of bacterial cells adhered to enamel surfaces in the Fluor defender®-treated group was significantly (*p* < 0.001) fewer than the Enamelast®-treated group by approximately 36.55% and 20.62% up to 48 and 72 h after exposure, respectively.

The noticeable low antimicrobial performance of Enamelast® could be attributed to its hydrophobic resinous content, which causes weak release of fluoride (Fernández et al. 2014). This assumption is consistent with Al Dehailan et al. who related the difference in composition of varnishes to their mechanism of action of releasing and deposition of fluoride on the outer layers of enamel lesions (Al Dehailan et al. 2016). Enamelast® contains higher concentration of fluoride, 22,600 ppm while Fluor defender® contains 1600 ppm fluoride. In this regard, no relation was detected between the fluoride concentration in the varnish and the fluoride release or its antimicrobial effect. This is consistent with the results obtained by Bolis et al. who compared different brands of varnish and found that Duraphat® varnish released the lowest and MI varnish™ the highest amount of fluoride while enamel fluoride uptake by both materials was not statistically different (Bolis et al. 2015). Additionally, lower viscosity of Fluor defender® than that of Enamelast® may have promoted greater release of fluoride with its antimicrobial effect. According to Carvalho et al., the lower viscosity of certain varnishes may boost stronger retention on enamel, provide greater contact, and allow greater release of fluoride (Carvalho et al. 2015).

Fluor defender® contains 0.1% fluorosilane in its formulation (a polyurethane-based compound) that may act by inhibiting the adhesion of *Streptococcus mutans* cells to the enamel surface and promoting fluoride release which inhibits demineralization (Baygin et al. 2014; Byeon et al. 2016; Punathil et al. 2018) and promotes remineralization (Yadav et al. 2019). Moreover, the protective quality of Fluor defender® is also attributed to the mechanical barrier provided by preventing direct contact of acids on the surface. The anti-streptococcal biofilm activity of Fluor defender® is basically linked to the fluoride incorporation into the crystalline lattice of enamel and formation of calcium fluoride after 24 h (Harding et al. 1994; Seppä 2004). A previous study by Erdem et al. reported that Fluor Protector® showed a better antibacterial effect when compared to Bifluoride 12 varnish. Although Bifluoride 12 had higher content of fluoride, they attributed the results to the Fluor Protector® silane content (Erdem et al. 2012). The latter has similar polyurethane-based compound; difluorosilane and a similar low fluoride concentration to Fluor defender®. The implication of this study supports the view that the higher antibacterial activity of Fluor defender® is attributed to its formulation. Additionally, Bezerra et al. (2022) studied the anti-cariogenic effect of Fluor Protector®, hybrid coatings, and a combination of stannous chloride and sodium fluoride using confocal microscopy. They reported that Fluor Protector® showed greater protection against *Streptococcus mutans* UA159 on bovine enamel (Bezerra et al. 2022). On the other hand, the fluoride content in Enamelast® may have hindered the effect of xylitol and consequently reduced the defensive effects of the varnish as compared to Fluor defender® (Cardoso et al. 2014; Mohd Said et al. 2017).

Some mandatory limitations were encountered in the current study. Since the caries process has a multifactorial nature, we could not cover all its aspects in our study. *Streptococcus mutans* was chosen in our model as it represents the primary source of caries initiation. We believe that a significant antimicrobial effect against *Streptococcus mutans* was obtained by Fluor defender®. However, a cariogenic challenge is recommended using other types of cariogenic flora such as lactobacilli. The lack of acquired salivary pellicle formation is another limitation of the study which would have influenced the interaction between fluoride and minerals on enamel surface (Souza et al. 2010). Moreover, polishing the specimens might have affected the varnish’s surface retention compared with clinical conditions (Rios et al. 2006) though it was imperative for standardization of specimens. Autoclaving of disk models was another limitation of the current study, but likewise it was a mandatory procedure in the methodology.

## Conclusions

The results of the present in vitro study in primary teeth have shown a significant difference between Fluor defender® and Enamelast® fluoride varnishes (*p* < 0.001) with respect to their antimicrobial efficacy against *Streptococcus mutans* biofilm. The antimicrobial activity of the Fluor defender® was greater than the Enamelast® varnish and consequently, the null hypothesis was rejected. Larger-scale in vitro and clinical studies should be performed to further verify these results. Fluor defender® seems like a promising antibacterial agent to be used for the primary dentition. Therefore, it is recommended to be incorporated in a preventive program for pediatric dental patients especially those with high risk for developing caries lesions.

## Data Availability

The datasets generated and analyzed during the current study are available from the corresponding author upon reasonable request.

## Abbreviations

dmft index: Decayed, missed, filled, teeth index
SEM: Scanning electron microscopy
ECC: Early childhood caries
PBS: Phosphate-buffered saline
