# Correction to: Evaluation of the antibacterial activity of Enamelast^®^ and Fluor defender^®^ fluoride varnishes against *Streptococcus mutans* biofilm: an in vitro study in primary teeth

**DOI:** 10.1007/s40368-023-00855-6

**Published:** 2023-12-23

**Authors:** M. A. Matar, S. S. Darwish, R. S. Salma, W. A. Lotfy

**Affiliations:** 1 Pediatric and Community Dentistry Department, Faculty of Dentistry, Pharos University in Alexandria, Alexandria, Egypt; 2 Pediatric Dentistry Department, College of Dentistry El Alamein, Arab Academy for Science, Technology and Maritime Transport (AAST), Alamein, Egypt; 3 Microbiology Department, Faculty of Dentistry, Pharos University in Alexandria, Alexandria, Egypt

**Correction to: European Archives of Paediatric Dentistry (2023) 24:549–558** 10.1007/s40368-023-00811-4

After publication in volume [24], issue [5], pages [549–558] the Figures of Scanning Electron Microscopy (3I, 4A, 4I, 5A, 5B, 5C, and 5I) in the original version of this article has been replaced.

The corrected version are given below:
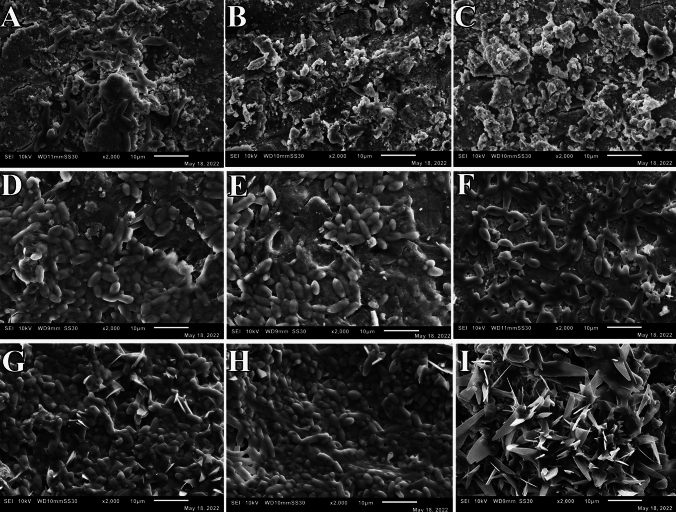

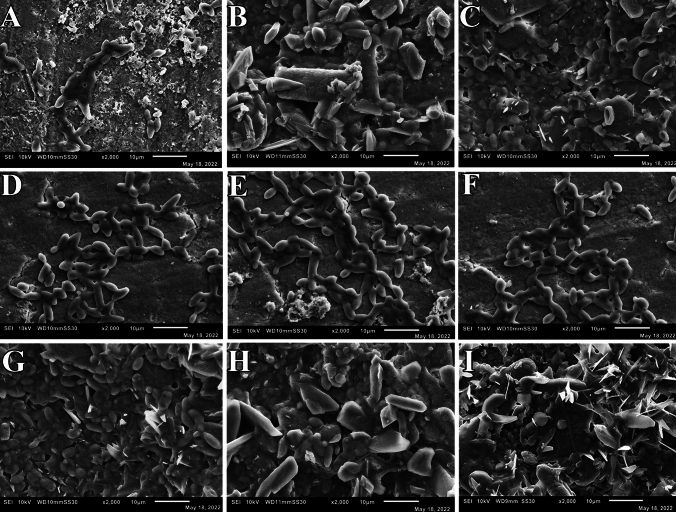

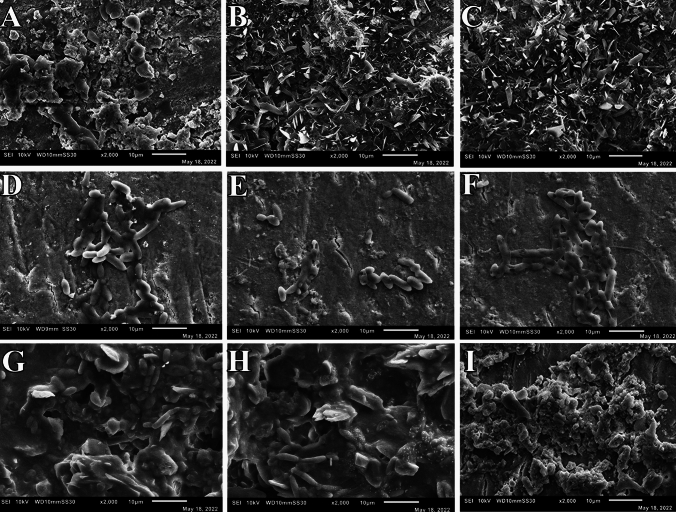


The original article has been updated accordingly.

